# Leveraging Autofluorescence for Tumor Detection, Diagnosis, and Accurate Excision with Surgical Margin Assessment in Tumor Excision

**DOI:** 10.3390/dj13010010

**Published:** 2024-12-26

**Authors:** Antonis Perdiou, Ramona Dumitrescu, Daniela Jumanca, Octavia Balean, Ruxandra Sava-Rosianu, Serban Talpos, Dacian Virgil Lalescu, Atena Galuscan

**Affiliations:** 1 Faculty of Dental Medicine, “Victor Babes” University of Medicine and Pharmacy Timisoara, Eftimie Murgu Square 2, 300041 Timisoara, Romania; antonis.perdiou@umft.ro (A.P.); dumitrescu.ramona@umft.ro (R.D.); jumanca.daniela@umft.ro (D.J.); sava-rosianu.ruxandra@umft.ro (R.S.-R.); galuscan.atena@umft.ro (A.G.); 2 Translational and Experimental Clinical Research Center in Oral Health (TEXC-OH), 14A Tudor Vladimirescu Ave., 300173 Timisoara, Romania; 3 Discipline of Oral and Maxillo-Facial Surgery, Faculty of Dental Medicine, “Victor Babes” University of Medicine and Pharmacy Timisoara, Eftimie Murgu Square 2, 300041 Timisoara, Romania; talpos.serban@umft.ro; 4 Department of Food Science, Faculty of Food Engineering, University of Life Sciences “King Mihai I” from Timisoara, 119 Calea Aradului Street, 300645 Timisoara, Romania; lalescu@usvt.ro

**Keywords:** autofluorescence, oral cancer, prevention, margins of tumoral excision

## Abstract

**Background/Objectives:** Oral cancer ranks among the top ten cancers globally, with a five-year survival rate below 50%. This study aimed to evaluate the effectiveness of autofluorescence-guided surgery compared to standard surgical methods in identifying tumor-free margins and ensuring complete excision. **Methods:** A prospective cohort of 80 patients was randomized into two groups: the control group underwent excision with a 10 mm margin based on clinical judgment, while the experimental group used autofluorescence guidance with a 5 mm margin beyond fluorescence visualization loss. Autofluorescence imaging was performed using the OralID device, which employs a 405 nm excitation laser to detect abnormal tissue. Ethical approval was obtained from the “Spitalul Clinic Municipal de Urgență Timișoara” Ethics Committee (approval number 08/26.02.2021), and the trial was registered at the University of Medicine and Pharmacy Timisoara (trial no. 59/25.11.2021). A double analysis was conducted: a primary analysis of the full cohort and a subgroup analysis focusing on squamous cell carcinoma (control: *n* = 19; experimental: *n* = 24). Histopathological analysis was the gold standard for margin evaluation, with margins coded as tumor-free margins (0), close (1), or infiltrated (2). **Results:** Statistically significant differences were observed in tumor-free margins between the control (73.17%) and experimental (97%) groups (*p* = 0.003). Subgroup analysis for SCC showed no significant difference (control: 84.21%; experimental: 95.83%; *p* = 0.306). Tumor location also differed significantly *(p* = 0.011), while other baseline variables, such as tumor type and patient characteristics, showed no significant differences. **Conclusions:** Autofluorescence-guided surgery improves the detection of tumor-free margins and may serve as an effective adjunct in oral cancer management. Larger studies are recommended to confirm these findings.

## 1. Introduction

Oral cancer is a major global public health issue, with dental practitioners playing a critical role in its early detection and management. It is among the top ten cancers by frequency, and despite advances in research and treatment, survival rates have not improved significantly in recent years, representing a continuing challenge to the biomedical scientific community [1].

Oral malignant tumors represent the 16th most common cancer in the world, having 177,757 deaths annually and, according to the WHO, as of 2020, 377,713 new cases [2,3].

The World Health Organization (WHO) emphasizes the importance of early detection in reducing the burden of oral cancer. Early detection is crucial for assessing the risks of malignant transformation, yet over 60% of oral cancer cases are diagnosed at advanced stages (III and IV), often due to small, asymptomatic lesions being overlooked [4].

Efficient diagnostic tools could enable clinicians to detect potentially malignant lesions earlier, thus reducing morbidity and mortality, improving patients’ quality of life, and lessening the financial impact of the disease [5].

Oral cancer is often preceded by a pre-malignant phase identifiable through visual inspection, offering opportunities for early intervention [6]. A majority (90%) of oral cancers originate in the epithelial cells of the oral cavity, with oral squamous cell carcinoma (SCC) being the most common type. The other 10% of malignant tumors comprise tumors of the salivary glands, melanomas, and lymphomas [7]. In Romania, oral malignant tumors are prevalent, with approximately 2388 new cases annually [8]. Although oral cancer may be preventable by reducing external high-risk factors like smoking, consuming alcohol, chewing tobacco, and sun exposure, the five-year survival rate is still below 50% [9,10]. The most significant prognostic factor for survival is the complete surgical removal of the tumor, as incomplete resection is a leading cause of death in oral cancer patients [11]. Surgery stands as the primary treatment for oral cancer, emphasizing the critical role of radical tumor resection in achieving recurrence-free, disease-free, and overall survival. However, 30–85% of surgeries result in inadequate resection margins, especially in deep areas, leading to a high recurrence rate of 10–30% [12]. These tumors are irregular in shape, having later extensions and different depths, making it difficult to completely remove them with a high rate of recurrence, i.e., 10–30% [13]. Still, there is no agreement on where the surgical clearance should be placed [14,15], and, even now, there is no global consensus about oral cancer screening.

New diagnostic techniques, such as toluidine blue staining and autofluorescence, are under study to aid early detection. Toluidine blue, a cationic metachromatic dye, stains pre-malignant and malignant lesions royal blue, aiding visual identification [16]. Chemiluminescence and autofluorescence imaging are additional tools that show promise in distinguishing normal from abnormal tissues by highlighting cellular changes associated with cancer, such as increased nuclear content and decreased stromal collagen fluorescence [17,18,19].

**Figure 1:**
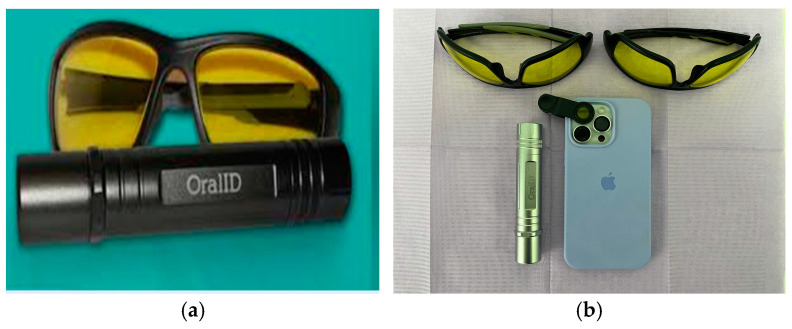
OralID device and protective glasses for autofluorescence imaging: (**a**) OralID device and protective glasses [20]. (**b**) Complete setup, including protective glasses, OralID flashlight, and smartphone attachment for imaging.

Autofluorescence devices, such as OralID, offer a non-invasive approach to detecting dysplastic or cancerous tissues, helping clinicians improve diagnostic accuracy and lesion management [20]. Studies show that combining these tools with conventional examination methods can significantly enhance the visualization of lesions and improve diagnostic accuracy [21,22]. Autofluorescence of the oral mucosa is influenced by the anatomical location and type of lesions present. In normal mucosa, fluorescence in the ultraviolet (UV) and visible spectrum is predominantly generated by collagen in connective tissue [21]. Normal mucosa, when excited with ultraviolet light, gives a light green color [22] (Figure 1), the epithelium shows reduced autofluorescence, mainly attributable to mitochondrial nicotinamide adenine dinucleotide dehydrogenase (NADH) and flavin adenine dinucleotide (FAD) in the basal cells of the epithelium. Keratin in the epithelium also contributes to fluorescence. In neoplasia, loss of stromal collagen occurs, implying a decrease in autofluorescence due to disruption of the distribution of the endogenous fluorophores mentioned above [23]. Some studies have shown that disruption of these elements shows not only loss of fluorescence but also hyper fluorescence [18]. Autofluorescence has also proven effective in detecting non-melanoma skin cancers, such as basal cell carcinoma (BCC) and squamous cell carcinoma (SCC), by identifying histopathological changes that reduce autofluorescence intensity compared to healthy tissues [21,22]. Epithelial dysplasia accentuates mitochondrial fluorescence of the epithelium. Inflammatory lesions are characterized by loss of autofluorescence in both epithelial and stromal cells [21].

Effective identification of malignant or pre-malignant lesions is essential for ensuring proper surgical excision with safe margins to avoid recurrence. The recurrence rate of SCC after surgery can be as high as 25–60%, depending on the stage of cancer at diagnosis [24].

Autofluorescence has emerged as a promising non-invasive diagnostic tool in the early detection and management of non-melanoma skin cancers, including BCC and SCC. Studies have demonstrated that alterations in autofluorescence intensity are associated with histopathological changes in cancerous tissues, such as hyperkeratosis, neoangiogenesis, and fibrosis, which contribute to a significant reduction in autofluorescence intensity compared to healthy tissues. In BCC and SCC, the mean autofluorescence intensity ratio is approximately four times lower than in healthy skin, suggesting that autofluorescence can be instrumental in distinguishing between malignant and normal tissues. This technique offers the potential to improve the accuracy of tumor margin assessment during surgical excision, thereby reducing the risk of recurrence and improving patient outcomes [25].

In the review by Bashkatov, Genina, and Tuchin (2011) that investigates the optical properties of skin, subcutaneous, and muscle tissues, it is noted that squamous cell carcinoma (SCC) and basal cell carcinoma (BCC) exhibit distinct autofluorescence patterns. SCC generally demonstrates higher autofluorescence intensity, largely attributed to increased keratin content and metabolic activity, whereas BCC shows lower autofluorescence due to its unique tissue architecture and reduced production of autofluorescent biomolecules [26].

Correct identification of malignant or pre-malignant lesions is essential for diagnostic and surgical excision of the lesion with the safety infiltrated margins to avoid recurrence. Camisasca et al. reported that patients had only a 30% five-year survival rate after recurrence of SCC and a 92% five-year survival rate in patients without recurrence [27]. SCC recurrence after surgical removal was found to be 25–30% in early-stage SCC and 50–60% in late-stage SCC [28]. 

Traditional methods for diagnosing and evaluating tumor margins, such as visual inspection and white light, although widely used, present significant limitations, including variability depending on the clinician’s experience and difficulty in distinguishing healthy tissues from tumorous ones in complex cases. Autofluorescence, as a diagnostic method, offers a distinct advantage due to its ability to highlight abnormal structures through fluorescence loss. This facilitates more accurate identification of malignant lesions and could provide a faster and more precise method for evaluating tumor margins compared to traditional methods [13].

This study aims to investigate the effectiveness of autofluorescence devices in more accurately identifying free surgical margins in malignant lesions, compared to conventional methods using white light. The goal is to demonstrate that autofluorescence can facilitate the determination of minimum safety margins without compromising the completeness of tumor excision.

## 2. Materials and Methods

This prospective cohort study included a total of 86 patients who presented to the “Spitalul Clinic Municipal de Urgență Timișoara”, at the Department of Oral and Maxillofacial Surgery, between 1 January 2022 and 1 May 2023. Of the 86 eligible patients, 6 declined to participate, leaving a final study population of 80 patients. All patients underwent an initial clinical consultation, followed by further diagnostic evaluations.

This study was conducted in compliance with the principles of the Declaration of Helsinki. Ethical approval was obtained from the Ethics Committee of the “Spitalul Clinic Municipal de Urgență Timișoara” (approval number 08/26.02.2021), and the trial was registered at the University of Medicine and Pharmacy Timisoara (trial no. 59/25.11.2021). All participants provided written informed consent after receiving detailed information about the study’s purpose, procedures, potential risks, and benefits.

Additional details, including supporting videos, are provided in the Supplementary Materials.

### 2.1. Inclusion and Exclusion Criteria

Patients were eligible for inclusion if they presented with primary tumors in the oral and maxillofacial region and had not received prior treatment (chemotherapy, radiotherapy, or surgery) for head or neck malignancies. The exclusion criteria included patients who did not provide informed consent to participate, patients with a history of prior malignancies, and those with any ongoing malignancy treatments.

### 2.2. Clinical and Diagnostic Evaluations

For each patient, a comprehensive medical history was recorded, including lifestyle factors such as smoking (both current and former smokers) and daily alcohol consumption. Additionally, comorbidities such as arterial hypertension and diabetes mellitus were noted.

All patients underwent a detailed clinical examination, including tactile and visual assessments. Tumor evaluation was conducted using computed tomography (CT) scans of the head and neck region. All patients underwent the same preoperative evaluation, including thoracic X-ray, cardiology consultation, and blood tests. Patients with findings indicating significant risks for surgery were excluded and accounted for in the analysis. Blood tests were conducted, including a complete blood count, coagulation profile, liver function tests, glucose levels, and kidney function tests (glomerular filtration rate).

### 2.3. Study Groups and Randomization

The 80 participants were randomly assigned into two groups. Randomization was conducted using a computer-generated sequence to ensure unbiased allocation of patients into the two groups, with stratification based on tumor size and smoking status. Allocation was concealed using sealed, opaque envelopes, which were opened only after patient consent was obtained.

- Control group (*n* = 41): This group underwent tumor excision following standard surgical protocols, which included excision based on clinical judgment, with margins of 10 mm from the visible edge of the tumor.
- Experimental group (*n* = 39): In this group, tumor excision was guided by autofluorescence technology, where tumor margins were determined using a fluorescence device. The excision margins were set at 5 mm beyond the fluorescence device-determined tumor edge.

Of the 80 patients initially enrolled, no patients were lost to follow-up. All analyses were performed on an intention-to-treat basis, including all patients as randomized, regardless of protocol deviations.

### 2.4. Surgical Procedure

After inspection and tactile examination, the examiner established the tumor margins, with the lesion excised either 5 mm beyond the fluorescence device (FD)-determined margins or 10 mm outside the clinically visible tumor. In both groups, the excision was performed under general anesthesia in the operating room. In the control group, intraoperative assessment of margins was conducted using clinical judgment, and additional tissue was excised if margins appeared compromised. Surgeons were blinded to the group assignment of the patients, and in cases of positive margins, re-excision was performed. In the experimental group, fluorescence-guided excision was used in conjunction with clinical judgment, with a minimum 5 mm margin beyond the fluorescent boundary. The excision outline was marked using an Easimark modern regular tip marker (Leonhard Lang, now Skintact, Fannin, Ireland). The surgical procedure was carried out using a scalpel, and electrocautery was employed to control intraoperative and postoperative bleeding. Resorbable sutures were used for the subcutaneous tissues, while non-resorbable polyamide sutures were applied to the superficial layers.

### 2.5. Postoperative Histopathological Examination

All excised tissues were sent for histopathological analysis, which served as the gold standard for confirming the diagnosis of malignancy and determining the adequacy of the excision margins. Tumors were staged based on the 8th edition of the Union for International Cancer Control (UICC)’s *TNM Classification of Malignant Tumors* [29]. The histopathological examination of resected margins was conducted by pathologists who were blinded to the group assignment, ensuring unbiased assessment of the presence of residual tumor cells.

To evaluate the excision margins, the following coding system was applied based on histopathological analysis:

Code 0: Tumor-free margins—no residual tumor cells present.
Code 1: Close margins—residual tumor cells located within 1 mm of the surgical margin.
Code 2: Infiltrated margins—residual tumor cells present at the surgical margin.

This coding system was uniformly applied to all excised specimens in both study groups to ensure consistency in the analysis of surgical outcomes

#### 2.5.1. OralID (Forward Science, Houston, TX, USA)

The OralID device, developed by Forward Science, is a compact, portable, battery-operated tool designed for oral mucosa screening (Figure 1a,b). Unlike traditional methods, this device does not require any consumables or mouth rinses with dye, offering a more convenient and efficient alternative. It emits blue light within the 435–460 nm wavelength range, and the kit comes with two pairs of specially designed yellow-lensed eyewear. These lenses allow the user to detect autofluorescence in the oral tissues. Healthy mucosa retains a green appearance when exposed to the blue light, a phenomenon referred to as fluorescence visualization retention. In contrast, abnormal, dysplastic, or cancerous tissues lose this green appearance, instead showing up as dark patches, a condition known as fluorescence visualization loss [30,31]. Operators (A.P and S.T.) were trained in a standardized protocol for device usage, and the technology has been validated in previous studies for detecting tumor margins with high sensitivity [30].

The patients were asked to rinse their mouths with water to remove the debris. Toothbrushing was avoided before the procedures to avoid any bleeding on site, which interferes with the correct fluorescence of the tissues since blood appears as dark areas.

**Figure 2:**
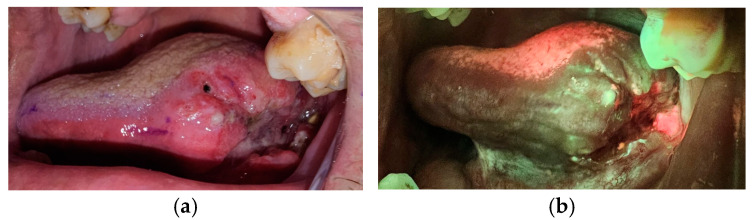
Tongue tumor. (**a**) Image taken in normal light. (**b**) Image taken with autofluorescence device.

All the lesions underwent excisional biopsies using the margins autofluorescence identified as free margins (Figure 2). All biopsies were oriented and sent for histopathological examination.

To account for the heterogeneity between tumor types, patients were categorized based on their histopathological diagnosis into SCC and BCC groups. As noted in the review by Bashkatov, Genina, and Tuchin (2011), SCC and BCC exhibit distinct autofluorescence patterns, with SCC showing higher autofluorescence intensity due to increased keratin content and metabolic activity, while BCC has lower autofluorescence because of its unique tissue structure and reduced production of autofluorescent biomolecules [26]. Given these differences in biological behavior and autofluorescence properties, a separate statistical analysis was conducted specifically for SCC to ensure the accuracy of the clinical outcomes related to this tumor type.

#### 2.5.2. Statistical Analysis

All statistical analyses and graphic representations were performed using R (version 4.2.2). Spearman’s rank correlations were computed to assess the relationships between the variables. Key patient characteristics, such as tumor size, smoking history, and comorbidities (e.g., diabetes and hypertension), were compared between the groups to ensure balance. Any significant differences between groups were adjusted for in the final analysis.

Fisher’s exact test and effect size analysis were employed to determine statistically significant differences between the experimental group and the control group.

Two levels of analysis were performed in this study to ensure a comprehensive evaluation of the autofluorescence technology:

Primary analysis: This analysis included the entire cohort of 80 patients, comprising both squamous cell carcinoma (SCC) and basal cell carcinoma (BCC) cases. The goal was to evaluate the overall effectiveness of autofluorescence-guided surgery in improving margin detection.

Secondary analysis: To assess the specific impact on the most prevalent oral malignancy, a subgroup analysis was conducted exclusively for patients diagnosed with SCC. This subgroup consisted of 43 patients: 19 in the control group and 24 in the experimental group.

This two-tiered approach allowed for both a broad evaluation of the technology and a more focused investigation of its role in SCC management.

To further analyze group differences in tumor margins, a logistic regression model was applied. The dependent variable was the presence of tumor-free margins (binary: yes/no), and the primary independent variable was group assignment (experimental vs. control). Potential confounders, including tumor location and size; smoking status; and comorbidities, such as diabetes and hypertension, were included as covariates in the model. Adjusted odds ratios (ORs) with 95% confidence intervals (CIs) were reported to evaluate the association.

## 3. Results

This study had two types of malignant tumors: squamous cell carcinoma (SCC) and basal cell carcinoma (BCC). In the control group, 17.07% of the patients (*n* = 7) were diagnosed, after histopathological examination, with basal cell carcinoma (BCC) and 82.93% (*n* = 34) with squamous cell carcinoma (SCC), whereas in the experimental group, 12.82% (*n* = 5) were diagnosed with BCC and 87.18% with SCC (*n* = 34). Fisher’s exact test *(p* = 0.757, Cramer’s V = 0.1746) shows us that there are no statistically significant differences between the experimental group and the control group with respect to the type of carcinoma. Also, 75.61% (*n* = 31) of the patients in the control group were males, and 24.39% (*n* = 10) were females, whereas 74.36% (*n* = 29) were males and 25.64% (*n* = 10) were females in the experimental group, respectively. Fisher’s exact test (*p* = 1; Cramer’s V = 0.0144) shows us that there are no statistically significant differences between the experimental group and the control group with respect to the sex of the patients (Table 1).

Regarding the localization of the tumor, in the control group, 29.27% (*n* = 12) were lower-lip tumors, 24.39% (*n* = 10) were upper-lip tumors, and 46.34% (*n* = 19) were tongue and floor-of-the-mouth tumors. In the experimental group, 5.13% (*n* = 2) were lower-lip tumors, 30.77% (*n* = 12) were tumors of the upper lip, jugal mucosa tumors accounted for 7.69% (*n* = 3), 53.85% (*n* = 21) were tongue and floor-of-the-mouth tumors, and, lastly, 2.56% (*n* = 1) signified a mandibular tumor. Fisher’s exact test (*p* = 0.011; Cramer’s V = 0.3772) shows statistically significant differences between the experimental group and the control group with respect to the location of the patients’ tumors (Table 1).

Considering the tumoral size, Fisher’s exact test (*p* = 0.3; Cramer’s V = 0.1746) showed no statistical significance between the two groups. In the control group, 2.4% (*n* = 1) of the patients were coded as T1, 21.95% (*n* = 9) of the patients were coded as T2, 46.34% (*n* = 19) of the patients were coded as T3, and, lastly, 29.27% (*n* = 12) of the patients were coded as T4. The experimental group comprised 5.13% (*n* = 2) of the patients who were coded as T1, 30.77% (*n* = 12) of the patients who were coded as T2, 51.28% (*n* = 20) of the patients who were coded as T3, and 12.82% (*n* = 5) of the patients who were coded as T4 (Table 1). 

Patients with comorbidities and associated diseases are presented in Table 2.

In the control group, 46.34% (*n* = 19) were with hypertension, and 53.66% (*n* = 22) were without hypertension, respectively, while in the experimental group, 61.54% (*n* = 24) were with hypertension and 38.46% (*n* = 15) were without hypertension. 

Examining alcohol intake revealed that, in the control group, 39.02% (*n* = 16) of the patients were users, while 60.98% (*n* = 25) were not, and in the experimental group, 61.54% (*n* = 24) were users, and 38.46% (*n* = 15) were not. 

In the control group, 17.07% (*n* = 7) were with type II diabetes and 82.93% (*n* = 31) were without type II diabetes, while in the experimental group, 17.95% (*n* = 7) were with type II diabetes and 82.05% (*n* = 32) were without type II diabetes. 

Fisher’s exact tests show us that there are no statistically significant differences between the experimental group and the control group with respect to arterial hypertension, alcohol intake, and type II diabetes.

As for smoking, in the control group, 56.1% (*n* = 23) were ever smokers, and 43.9% (*n* = 18) were not, while in the experimental group, 76.92% (*n* = 30) were ever smokers and 23.08% (*n* = 9) were not. Fisher’s exact test (*p* = 0.061) shows that there are not marginally statistically significant differences between the experimental group and the control group with respect to smoking criteria (Table 2).

The results revealed significant differences between the control and experimental groups in terms of free surgical margins and coding system classifications. In the experimental group, 97% (*n* = 38) of patients achieved free surgical margins, compared to 73% (*n* = 30) in the control group, while only 3% (*n*= 1) of the experimental group had no free margins, compared to 27% (*n* = 11) in the control group (*p* = 0.003; Cramer’s V = 0.3397). Similarly, the coding system showed that 77% (*n* = 31) of patients in the experimental group were classified as 0, reflecting the most favorable outcome, compared to 44% (N = 18) in the control group. In contrast, 21% (*n* = 7) and 3% (*n* = 1) of the experimental group were classified as 1 and 2, respectively, compared to 29% (*n* = 12) and 27% (*n* = 11) in the control group (*p* = 0.002; Cramer’s V = 0.3888). These findings highlight the superior outcomes observed in the experimental group for achieving both tumor-free surgical margins and favorable coding classifications (Table 3).

**Figure 3:**
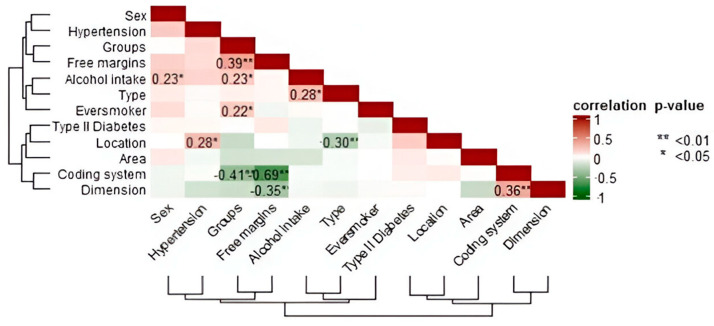
Correlation and *p*-value of all variables.

Spearman’s rank correlation matrix was calculated (see Figure 3). A statistically significant high negative correlation (*ρ* = −0.69 **; 95% CI [−0.81, −0.55]) was observed between margins and coding system, and a statistically significant low positive correlation (*ρ* = 0.23 *; 95% CI [0.00, 0.43]) between sex and alcohol intake. There were statistically significant high negative correlations between groups and coding system (*ρ* = −0.41 **; 95% CI [−0.56, −0.19]), between free margins and dimension of the tumor (*ρ* = −0.35 *; 95% CI [−0.52, −0.19]), and between type of tumor and localization (*ρ* = −0.30 **; 95% CI [−0.61, −0.26]). Statistically significant positive correlations also were found between the groups and free margins (*ρ* = 0.39 **; 95% CI [0.15, 0.48]) and between the dimension of the tumor and the coding system of the margins (*ρ* = 0.36 **; 95% CI [0.16, 0.56]).

In the experimental group, 97% of the patients achieved tumor-free margins, compared to 73.17% in the control group (Fisher’s exact test, *p* = 0.003). The calculated odds ratio was 13.93, indicating that the experimental group was approximately 14 times more likely to achieve tumor-free margins. Additionally, the effect size measured by Cramer’s V was 0.3397, suggesting a medium effect size.

A separate statistical analysis was conducted focusing on patients with oral squamous cell carcinoma due to its higher clinical importance. The control group included 19 patients, and the experimental group had 24. Among the control group, 73.68% (*n* = 14) were males and 26.32% (*n* = 5) were females, while in the experimental group, 75% (*n* = 18) were males and 25% (*n* = 6) were females, with no statistically significant difference between the groups (*p* = 1). Regarding tumoral excision margins, 84.21% (*n* = 16) of patients in the control group had tumor-free margins, compared to 95.83% (*n* = 23) in the experimental group. Infiltrated margins were observed in 15.79% (*n* = 3) of the control group and 4.17% (*n* = 1) of the experimental group, with no significant difference between groups (*p* = 0.306; Cramer’s V = 0.1498). Tumor localization showed that, in the control group, all tumors (100%, *n* = 19) were located on the tongue and floor of the mouth, while in the experimental group, 87.5% (*n* = 21) were located on the tongue and floor of the mouth, and 12.5% (*n* = 3) were jugal tumors, with no statistically significant difference (*p* = 0.243; Cramer’s V = 0.2437). Tumor size distribution also showed no significant differences (*p* = 0.325; Cramer’s V = 0.2716). In the control group, 5.26% (*n* = 1) were coded as T1, 10.53% (*n* = 2) as T2, 57.89% (*n* = 11) as T3, and 26.32% (*n* = 4) as T4. In the experimental group, 4.17% (*n* = 1) were T1, 33.33% (*n* = 8) were T2, 45.83% (*n* = 11) were T3, and 16.67% (*n* = 4) were T4. These findings indicate no statistically significant differences between the control and experimental groups in terms of sex, excision margins, tumor localization, or size (Table 4).

Regarding the margins of tumoral excision, 84% (*n* = 13) of patients in the control group had tumor-free margins, while 16% (*n* = 6) had infiltrated margins. In the experimental group, 96% (*n* = 23) of patients had tumor-free margins, and 4% (*n* = 1) had infiltrated margins. Fisher’s exact test (*p* = 0.306; Cramer’s V = 0.1987) indicated no statistically significant differences between the two groups with respect to tumor-free margins. Examining the coding system for excision margins, in the control group, 53% (*n* = 10) of patients were coded as 0, 16% (*n* = 3) as 1, and 32% (*n* = 6) as 2. In the experimental group, 71% (*n* = 17) were coded as 0, 25% (*n* = 4) as 1, and 4% (*n* = 1) as 2. Fisher’s exact test (*p* = 0.064; Cramer’s V = 0.3699) suggested a trend toward statistical significance, reflecting differences in the distribution of coding system categories between the experimental and control group (Table 5).

Comorbidities and associated diseases of the patients are present in the table below (Table 2). The table shows a comparison of comorbidities between the control and experimental groups. While there are no significant differences between the groups in terms of smoking (*p* = 0.521), alcohol intake (*p* = 0.132), or type II diabetes (*p* = 0.363), there is a statistically significant difference in the prevalence of arterial hypertension (*p* = 0.034), with 54.17% of patients in the experimental group having hypertension compared to only 21.05% in the control group. This suggests that arterial hypertension is more common in the experimental group (Table 6).

**Figure 4:**
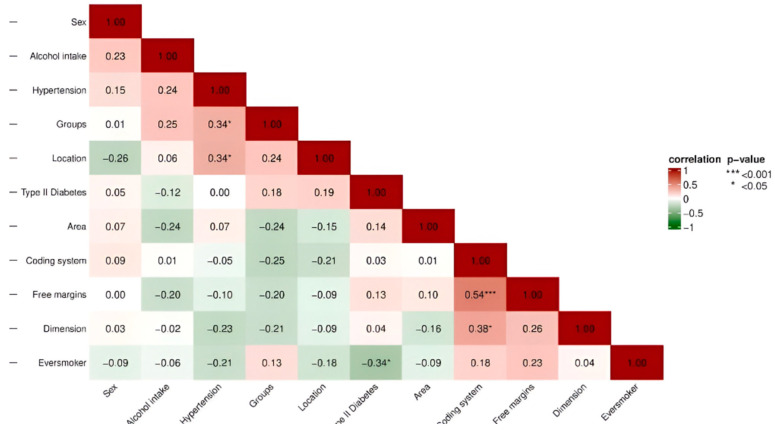
Correlation and *p*-value of all variables.

Spearman’s rank correlation matrix was calculated. A statistically significant high positive correlation (*ρ* = 0.54 ***; 95% CI [0.28, 0.72]) was observed between margins and coding system, and a statistically significant low positive correlation (*ρ* = 0.23; 95% CI [0.07, 0.51]) between sex and alcohol intake. There was a statistically significant low negative correlation between groups and coding system (*ρ* = −0.25, 95% CI [−0.53, −0.08]) and a significant low positive correlation between free margins and dimension of the tumor (*ρ* = 0.26 *, 95% CI [0.022–0.48). A statistically significant negative correlation was also found between the groups and free margins (*ρ* = −0.20; 95% CI [−0.44, −0.13]), and between the dimension of the tumor and the coding system of the margins, a significant positive correlation (*ρ* = 0.38 *; 95% CI [0.09–0.62]) was found (Figure 4).

The Spearman correlation analysis identified both high and low correlations among the studied variables. A statistically significant high positive correlation was observed between margins and coding system (*ρ* = 0.54 ***; 95% CI: [0.28, 0.72]), while a statistically significant low positive correlation was noted between sex and alcohol intake (*ρ* = 0.23 *; 95% CI: [0.00, 0.43]). Statistically significant low negative correlations were found between groups and coding system (*ρ* = −0.25; 95% CI: [−0.53, −0.08]) and between free margins and tumor dimension (*ρ* = −0.35 *; 95% CI: [−0.52, −0.19]). Additionally, a statistically significant high negative correlation was identified between type of tumor and localization (*ρ* = −0.30 **; 95% CI: [−0.61, −0.26]). Positive correlations were also observed, including a statistically significant correlation between groups and free margins (*ρ* = 0.39 **; 95% CI: [0.15, 0.48]) and between tumor dimension and the coding system of the margins (*ρ* = 0.36 **; 95% CI: [0.16, 0.56]). These findings provide detailed insights into the relationships between clinical and demographic variables in the study.

Logistic regression analysis was performed to model the likelihood of achieving tumor-free margins, adjusting for potential confounders. The experimental group was associated with higher odds of achieving tumor-free margins (OR = 1.75; 95% CI: [0.68, 4.51]), though this did not reach statistical significance. Tumor stage showed a slight positive association with tumor-free margins (OR = 1.13; 95% CI: [0.91, 1.41]), while comorbidities were negatively associated (OR = 0.48; 95% CI: [0.17, 1.35]); however, neither of these factors was statistically significant. These findings suggest that while the experimental group trends toward better outcomes, further research with larger sample sizes is needed to confirm these results.

## 4. Discussion

A significant factor contributing to the high mortality and morbidity rates associated with oral cancer prognosis is late detection and diagnosis. Delay in diagnosis of oral squamous cell carcinoma (SCC) is attributed to factors involving both the patient and the clinician. Cancer patients frequently require medical care at advanced stages due to lack of symptom awareness and insufficient understanding of the disease course. In addition, delays can be influenced by factors related to healthcare providers, further complicating timely diagnosis in SCC cases [32].

The rate of malignant transformation is subject to variations based on histologic grading, and the majority of such lesions may not advance to oral squamous cell carcinoma (SCC). Nevertheless, it remains crucial to identify and diagnose oral dysplastic lesions and early-stage SCC to enhance survival rates and prevent the need for extensive surgical interventions that might be associated with diagnostic delays [33].

The limitations of the human eye’s perception are particularly pronounced, especially when it comes to the wet and shiny mucosa, which introduces variability in reflection. The challenges associated with traditional oral examination (COE) have driven the exploration of alternative detection methods. One new approach involves the adoption of “optical biopsy systems”, which build on our understanding of the interaction between light and tissue. Autofluorescence, part of this innovative approach, relies on inherent fluorochromes present in the epithelium and submucosa to detect specific lesions.

The primary criterion for identifying tumorous tissue in this study was based on the loss of fluorescence (hypo-fluorescence) when compared to surrounding healthy tissue. Hyper-fluorescence was not deemed clinically useful, in line with the current understanding that it is the loss of fluorescence, rather than patterns of hyper- or hypo-fluorescence, which signals the presence of malignancy and residual tumor at the margins. This approach is consistent with prior research, which highlights that the breakdown of collagen and alterations in metabolic markers like NADH and FAD contribute to reduced fluorescence in cancerous tissues [13]. Therefore, our study focused on identifying these areas of fluorescence loss to guide surgical excision and ensure complete removal of malignant tissues.

The essential role of autofluorescence devices in detecting various lesions in the oral cavity, such as dental caries or soft tissue abnormalities (tumors), cannot be overestimated. Building on previous research, which has evaluated the effectiveness of laser fluorescence and light-induced fluorescence devices in improving diagnostic outcomes and early identification of lesions, this study highlights the importance of using innovative technologies for better oral health assessment and management [34].

In the oncological surgery area, the main goal is to obtain adequate resection margins to minimize the risk of recurrence and improve prognosis [12,35]. Both this research and other studies highlight the importance of proper evaluation of tumor excision margins.

Moro et al. conducted a study of 65 biopsies, of which only 31 were confirmed as true positives. Their focus on infiltrated margins revealed that, when subjected to a specific diagnostic method, 77.4% of these lesions had infiltrated margins. In comparison, visual inspection with the naked eye identified infiltrated margins in 67.7% of cases. Moro et al. concluded that there was no statistically significant disparity between the effectiveness of the specific diagnostic method and conventional visual inspection in assessing excisional margins [36]. In the present research, examination of tumor excision margins revealed distinct patterns between control and experimental groups. Of note, in the control group, 73.17% of patients were considered to have tumor-free margins, while 26.83% showed infiltrated margins. Notably, all patients in the experimental group showed tumor-free margins. The statistically significant differences observed between the control and experimental groups demonstrate the potential clinical utility of innovative diagnostic methods, as reflected in the superior results in the experimental group with tumor-free margins.

The study by Lajolo et al. (2022) analyzes the effectiveness of the autofluorescence device (Glasses for Oral Cancer—Curing Light Exposed—Screening, Pierrel S.p.A., Italy) in identifying oral lesions. Specificity and sensitivity of the device were reported at 48% and 66%, respectively, suggesting moderate performance in diagnosing these lesions. Positive and negative predictive values, recorded at 34% and 77%, respectively, indicate the usefulness of the autofluorescence device in combination with traditional methods of clinical examination and palpation of lesions [10]. In contrast, in the present study, the use of the autofluorescence device showed an increased success rate in identifying oral lesions. 

Head and neck cancer ranks as the sixth most prevalent form of human cancer, with oral cancer comprising 48% of the cases within the head and neck region [9].

The multifactorial nature of oral cancer involves multiple risk factors, with tobacco and alcohol use recognized as the main factors contributing to oral malignancy [37]. In addition, diabetes has emerged as another risk factor linked to the development of cancerous lesions in the oral cavity. A study exploring the association between oral cancer and type 2 diabetes identified this link in a small percentage of people, with 78 out of 379,138 (0.02%) people with type 2 diabetes reported to have oral cancer. Among people with type 2 diabetes, a higher prevalence and risk of oral cancer was observed in those who use tobacco (32.6%; OR = 2.52; 95% CI: 1.5–4.3), use alcohol (29.2%; OR = 2.01; 95% CI: 1.2–3.3), and have hypertension (23.9%; OR = 2.05; 95% CI: 1.2–3.6) and hypertriglyceridemia (24.7%; OR = 1.66; 95% CI: 1.01–2.7) [38]. Fisher’s exact tests in the context of the current study, however, show no statistically significant differences between the experimental and control groups in terms of hypertension, alcohol consumption, and type II diabetes. This suggests that the distribution of these factors is comparable between the two groups. 

The significant increase in the global mortality rate associated with lip and oral cavity cancers over the last three decades, with a 1.40-fold increase, is a worrying finding, highlighting the significant impact of these malignancies on global health. It is also noted that approximately one-third (30.5%) of deaths attributed to these cancers are still linked to tobacco use [39]. The global smoking trend is also reflected in the present study, where both the control and experimental groups are dominated by smokers.

Analysis of smoking habits between control and experimental groups revealed a notable prevalence, although it did not reach statistical significance (*p* = 0.061). The substantial proportion of smokers in both groups highlights the need to consider smoking habits as potential risk factors for oral cancer. This observation gains significance in the context of the documented increase in the incidence of oral cancer globally, mainly related to tobacco use. It is imperative to understand the impact of smoking given the critical role of early detection in increasing survival rates. Understanding how smoking habits might affect disease progression and response to treatment is essential to unravel the complexity of oral cancer etiology and management [40].

Oral cancer is a significant global health problem with a major impact on morbidity and mortality. The prevalence of this condition varies significantly around the world. Also, globally, the incidence of oral cancer is higher in men than in women, and the associated risk increases with age [41]. In our study, we found that the number of men in both groups (control and experimental) is higher than that of women, reflecting the general findings of the literature on the increased prevalence of oral cancer in men. 

Oral cancer can be found in various anatomical regions, although specific areas have a higher prevalence. The most frequent sites are the tongue and the floor of the mouth. In addition, it can affect other regions, such as the oral mucosa, retromolar area, gingiva, soft palate, and, more rarely, back of the tongue and hard palate [42]. These findings are in agreement with the results obtained, with the predominant distribution of tumor formations in the tongue and palate representing a significant proportion of 46.34% and 53.85%, respectively, in both the control and experimental groups.

SCC is the most common form of tumor in this region, accounting for 91% of all head and neck malignancies and 90% of oral malignancies. Although diagnosis and therapies have evolved, given new techniques and protocols, survival rates remain low [43]. According to the literature data and the results of the present study, SCC was present in a predominant percentage (82.93% in the control group and 87.18% in the experimental group), followed by SCB. Statistical analysis revealed no significant differences between the two groups, thus suggesting that the distribution of carcinoma types in the two groups was not significantly influenced by the use of autofluorescence compared to white light in our study.

Timely diagnosis and intervention play a key role in improving patient survival. Devices using autofluorescence technology for direct visualization of the oral cavity stand out as a potential tool for oral cancer detection or as an additional component in standard clinical examinations. Rana et al. (2012) used a protocol for a randomized control trial that observed 100% sensitivity in a combined diagnostic method using both a fluorescence device and a conventional examination, in contrast to 17% sensitivity using conventional examination alone [44]. Thus, in agreement with the literature, we can say that the role of devices using autofluorescence as an adjunct to conventional examinations offers a promising avenue for improving oral cancer detection, highlighting the need for a comprehensive diagnostic approach in clinical practice.

Vibhute et al. (2021), following the use of another fluorescent device in a small study of 30 patients, concluded that autofluorescence cannot completely replace conventional oral examination, but it should serve as a complementary method [45], as was proven in the present case, where the use of OralID, together with oral examination, lead to excision of the entire tumor formation in the experimental group. 

Some other studies reported results that were not that satisfactory. Awan et al. (2011) performed a study on oral potentially malignant disorders using Velscope and found 15.3% specificity [22]. One limitation of autofluorescence is what they called the umbrella effect. Hyperkeratotic tissue has an increased level of fluorophore, thus masking the neoplastic areas [46,47]. The question was raised as to how much the oral fluids influence the fluorescence readings. It was found out that an oral cavity rich in saliva gives no different readings, whilst where there is blood, the readings are different, since blood appears black under UVA light [48].

In this study, we conducted a separate statistical analysis specifically for patients with squamous cell carcinoma (SCC), given the distinct biological behavior of SCC compared to other tumor types. The results showed that 95.83% of patients in the experimental group had tumor-free margins, compared to 84.21% in the control group. However, Fisher’s exact test (*p* = 0.306) indicated no statistically significant difference between the two groups regarding the completeness of tumor excision. While this suggests that autofluorescence-guided surgery may improve margin clearance, the lack of statistical significance underscores the need for further research with larger sample sizes to fully establish the clinical utility of autofluorescence in SCC excision.

Additionally, the analysis of tumor location demonstrated no significant differences between the two groups (*p* = 0.243), with the majority of tumors in both groups located in the tongue and floor of the mouth. These findings are consistent with the known prevalence of SCC in these regions. However, autofluorescence-guided surgery showed promising results in identifying tumor margins, especially in high-risk locations.

Our findings demonstrated not only statistically significant differences between the experimental and control groups but also a clinically meaningful effect size. The odds ratio underscores the substantial advantage of autofluorescence-guided surgery in achieving tumor-free margins, while the Cramer’s V of 0.3397 confirms a medium effect size. These results emphasize the potential of autofluorescence technology to improve surgical outcomes in oral cancer treatment.

Logistic regression analysis revealed a trend toward higher odds of achieving tumor-free margins in the experimental group, though the association did not reach statistical significance. The influence of covariates, such as tumor size and comorbidities, underscores the multifactorial nature of surgical outcomes in oral cancer. These results highlight the potential benefit of autofluorescence-guided surgery, though larger studies are needed to confirm its effectiveness while accounting for confounding variables.

The observed statistical significance in tumor location between groups suggests that location may influence both imaging performance and surgical decision-making. Tumors in the tongue and floor of the mouth present unique challenges, as their anatomical complexity and proximity to critical structures can affect the effectiveness of autofluorescence imaging. Additionally, the variability in tissue structure and accessibility could impact the contrast and clarity of autofluorescence signals, potentially influencing surgical precision. Future research should investigate how specific tumor locations interact with autofluorescence technology to optimize its clinical application.

A study by Morikawa et al. (2019) demonstrated that using fluorescence visualization loss to guide tumor excision in early-stage squamous cell carcinoma (SCC) of the tongue resulted in improved local control rates and reduced recurrence [49]. In our study, the use of autofluorescence-guided excision similarly showed a higher percentage of tumor-free margins (95.83%) compared to conventional methods (84.21%). Although these differences were not statistically significant (*p* = 0.306), the results suggest that autofluorescence has potential clinical value in enhancing margin assessment and improving surgical outcomes.

The combination of clinical inspection and fluorescence visualization may provide a more accurate approach for identifying tumor margins, particularly in areas where traditional visual inspection is challenging.

In line with the findings of Tiwari et al. (2019), our study supports the use of autofluorescence as an adjunct to clinical oral examination for the detection and management of oral squamous cell carcinoma (OSCC). Tiwari et al. highlighted that autofluorescence enhances the ability to identify and visualize oral potentially malignant disorders by improving the detection sensitivity of lesions, especially when used alongside conventional examination techniques [24]. Similarly, in our study, autofluorescence-guided surgery led to improved visualization of tumor margins and a higher rate of tumor-free excision margins, though the statistical significance of these results was not reached.

The choice of using a 10 mm margin in the control group and a 5 mm margin beyond the autofluorescence boundary in the experimental group was intentional and reflects the study’s hypothesis. The control group followed conventional surgical protocols, where a 10 mm margin is often employed to account for uncertainties in visually assessing tumor boundaries. Conversely, the experimental group utilized autofluorescence technology to precisely identify tumor boundaries, allowing for a smaller safety margin of 5 mm beyond the fluorescence visualization loss. This approach was designed to test whether autofluorescence could provide equivalent or superior tumor clearance while preserving more healthy tissue. By comparing these two strategies, we aimed to demonstrate the potential of autofluorescence to refine surgical practices and reduce tissue loss without compromising oncological safety.

One of the key limitations of this study is the relatively small sample size, which may have affected the statistical power of the findings, particularly in demonstrating the significance of the results regarding autofluorescence-guided surgery. Additionally, while we focused on the use of autofluorescence to guide excision margins, this technique may have inherent limitations when dealing with hyperkeratotic or inflammatory tissues, which can interfere with fluorescence readings and potentially mask malignant areas. Another limitation is the inclusion of different tumor types, such as SCC and BCC, which have distinct autofluorescence patterns. This introduces variability in the findings, as the behavior and surgical management of these tumors differ.

While the sample size in this study was determined based on feasibility within the given timeframe and resources, we recognize that a larger sample could increase the statistical power for detecting smaller differences. Nevertheless, the significant difference observed in the primary outcome suggests that autofluorescence-guided surgery has a clinically meaningful impact on achieving tumor-free margins. Future research should aim to replicate these findings with larger sample sizes to confirm their generalizability and improve the robustness of the results.

While this study provides promising evidence that autofluorescence can improve margin detection in oral cancer surgery, its conclusions are limited by the relatively small sample size. Although the findings were statistically significant in several analyses, the small cohort size may reduce the generalizability of the results. Furthermore, subgroup analysis of squamous cell carcinoma (SCC) patients was constrained by the smaller sample within this specific group, which may have limited our ability to detect additional significant differences. Larger, multicenter studies with more diverse patient populations are necessary to confirm these preliminary findings and explore the broader applicability of autofluorescence-guided surgery in different clinical settings.

## 5. Conclusions

The autofluorescence properties of oral tissues should be more effectively leveraged through the development of innovative devices, including hands-free solutions, to facilitate intraoperative use, enhance excision accuracy, and minimize the occurrence of incomplete surgical margins. This study highlights autofluorescence as a valuable supplementary tool for guiding surgical excisions in oral cancer. By improving the precision of margin detection, autofluorescence has the potential to reduce recurrence rates. The positive outcomes observed in this study, where autofluorescence was employed to achieve complete tumor excision, indicate that it could serve as a significant complementary technique in reducing recurrence and improving survival rates in oral cancer patients.

## Figures and Tables

**Table 1:** Comparison of clinical and demographic characteristics between control and experimental groups.

Variables		*n* Control Group (%)	*n* Experimental Group (%)	*p*-Value
Type of malignant tumor	BCC	7 (17.07%)	4 (12.82%)	0.757
	SCC	34 (82.93%)	35 (87.18%)	
Sex	M	31 (75.61%)	35 (87.18%)	1
	F	10 (24.39%)	4 (12.82%)	
Tumoral Location	Lower Lip	12 (29.27%)	2 (5.13%)	0.011
	Upper Lip	10 (24.39%)	12 (30.77%)	
	Jugal		3 (7.69%)	
	Floor of the mouth	19 (46.24%)	21 (53.85%)	
	Mandible		1 (2.56%)	
TNM stage	T1	1 (2.44%)	2 (5.13%)	0.306
	T2	9 (21.95%)	12 (30.77%)	
	T3	19 (46.34%)	20 (51.28%)	
	T4	12 (29.27%)	5 (12.82%)	

**Table 2:** Comorbidities and associated diseases.

Comorbidities	Control Group		Experimental Group		*p*-Value
Smoking	Yes	No	Yes	No	0.061
	56.1% (*n* = 23)	43.9% (*n* = 18)	76.92% (*n* = 30)	23.08% (*n* = 9)	
Alcohol intake	39.02% (*n* = 16)	60.98% (*n* = 25)	61.54% (*n* = 24)	38.46% (*n* = 15)	1
Tye II diabetes	17.07% (*n* = 7)	82.93% (*n* = 31)	17.95% (*n* = 7)	82.05% (*n* = 32)	1
Arterial hypertension	46.34% (*n* = 19)	53.66% (*n* = 22)	61.54% (*n* = 24)	38.46% (*n* = 15)	0.187

**Table 3:** Comparison of free margins and coding system between control and experimental groups.

Variables		*n* Control Group (%)	*n* Experimental Group (%)
Free margins	Yes	30 (73%)	38 (97%)
	No	11 (27%)	1 (3%)
Coding System	0	18 (44%)	31 (77%)
	1	12 (29%)	7 (21%)
	2	11 (27%)	1 (3%)

**Table 4:** Comparison of demographics, tumor location, and tumor size between control and experimental groups in patients with squamous cell carcinoma.

Variables		*n* Control Group (%)	*n* Experimental Group (%)	*p*-Value
Sex	M	14 (73.68%)	18 (75%)	1
	F	5 (26.32%)	6 (25%)	
Location	Floor of the mouth	19 (100%)	21 (87.5%)	0.243
	Jugal		3 (12.5%)	
Size	T1	1 (5.26%)	1 (4.17%)	0.325
	T2	2 (10.53%)	8 (33.33%)	
	T3	11 (57.89%)	11(45.83%)	
	T4	5 (26.32%)	4 (16.67%)	

**Table 5:** Comparison of free margins and coding system between control and experimental groups in patients with oral squamous cell carcinoma.

Variables		*n* Control Group (%)	*n* Experimental Group (%)	*p*-Value
Free margins	Yes	13 (84%)	23 (96%)	0.306
	No	6 (16%)	1 (4%)	
Coding System	0	10 (53%)	17 (71%)	0.064
	1	3 (16%)	4 (25%)	
	2	6 (32%)	1 (4%)	

**Table 6:** Comorbidities and associated diseases.

Comorbidities	Control Group		Experimental Group		*p*-Value
Smoking	Yes	No	Yes	No	0.521
	57.89% (*n* = 13)	42.11% (*n* = 6)	70.83% (*n* = 17)	29.17% (*n* = 7)	
Alcohol intake	42% (*n* = 8)	58% (*n* = 11)	67% (*n* = 16)	33% (*n* = 8)	0.132
Type II diabetes	5.26% (*n* = 1)	94.74% (*n* = 18)	16.67% (*n* = 4)	83.33% (*n* = 20)	0.363
Arterial hypertension	21.05% (*n* = 4)	78.95% (*n* = 15)	54.17% (*n* = 13)	45.83% (*n* = 11)	0.034

## Data Availability

The primary/raw data, from which the figures, graphs, and tables were generated, are available upon request from the corresponding author. These are the data we referred to for Supplementary Data, and they can be provided if requested. There are no ethical reasons restricting the data.

## Supplementary Materials

The following supporting information can be downloaded at: https://www.mdpi.com/article/10.3390/dj13010010/s1, Video S1: Clinical Presentation of a Tongue Tumor With OralID; Video S2: Clinical Presentation of a Tongue Tumor Without OralID; Figure S1: Medical Procedure Using OralID Technology1; Figure S2: Medical Procedure Using OralID Technology2.
