# Supplementary figures and images for: Leveraging Autofluorescence for Tumor Detection, Diagnosis, and Accurate Excision with Surgical Margin Assessment in Tumor Excision
Source: Dent J (Basel). 2024 Dec 26;13(1):10. doi: 10.3390/dj13010010 (PMC11763627; doi:10.3390/dj13010010)

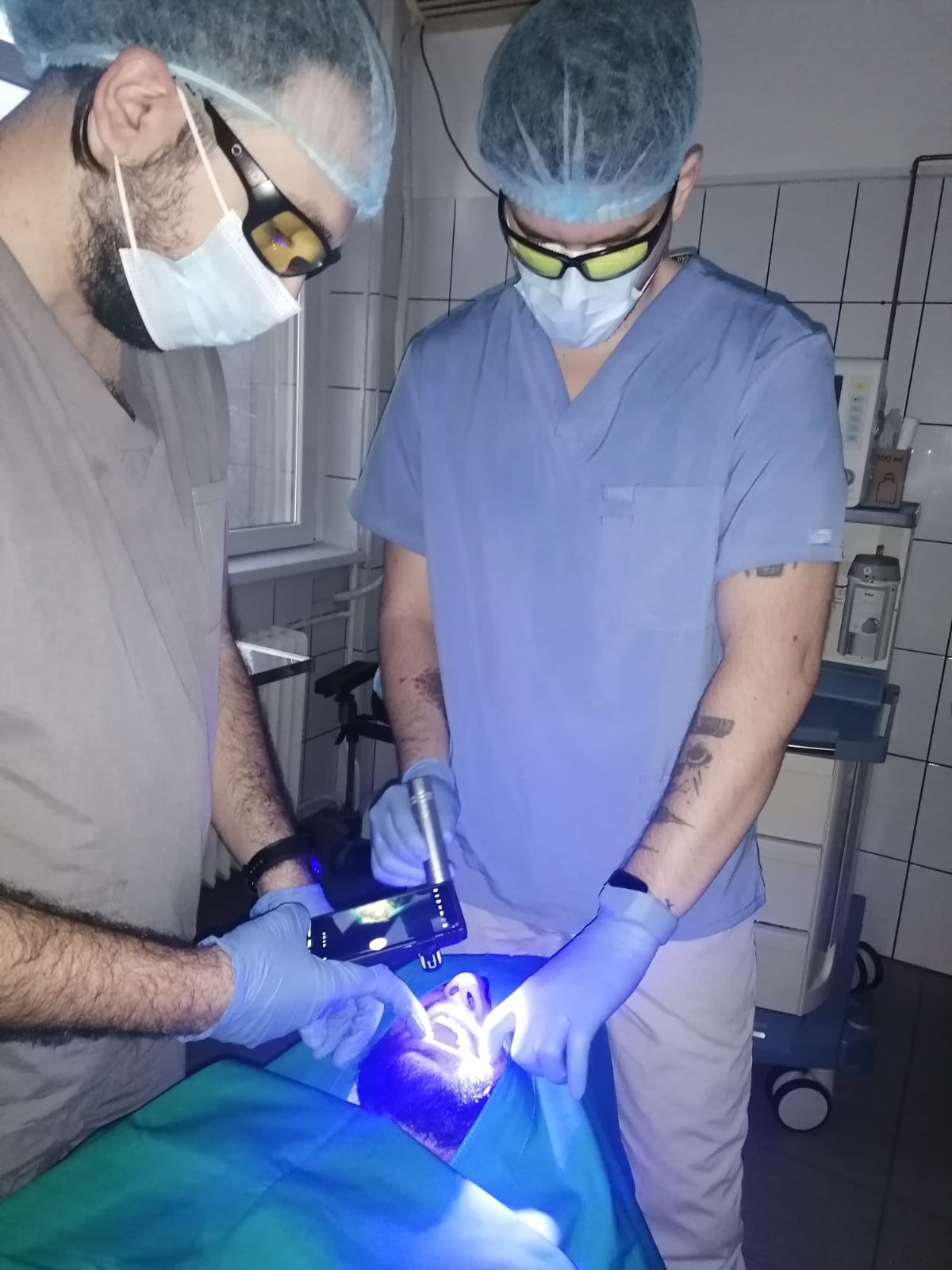

Supplement: Supplementary file 1 [file dentistry-13-00010-s001.zip › dentistry-3245039-supplementary/Medical Procedure Using OralID Technology1.jpeg]

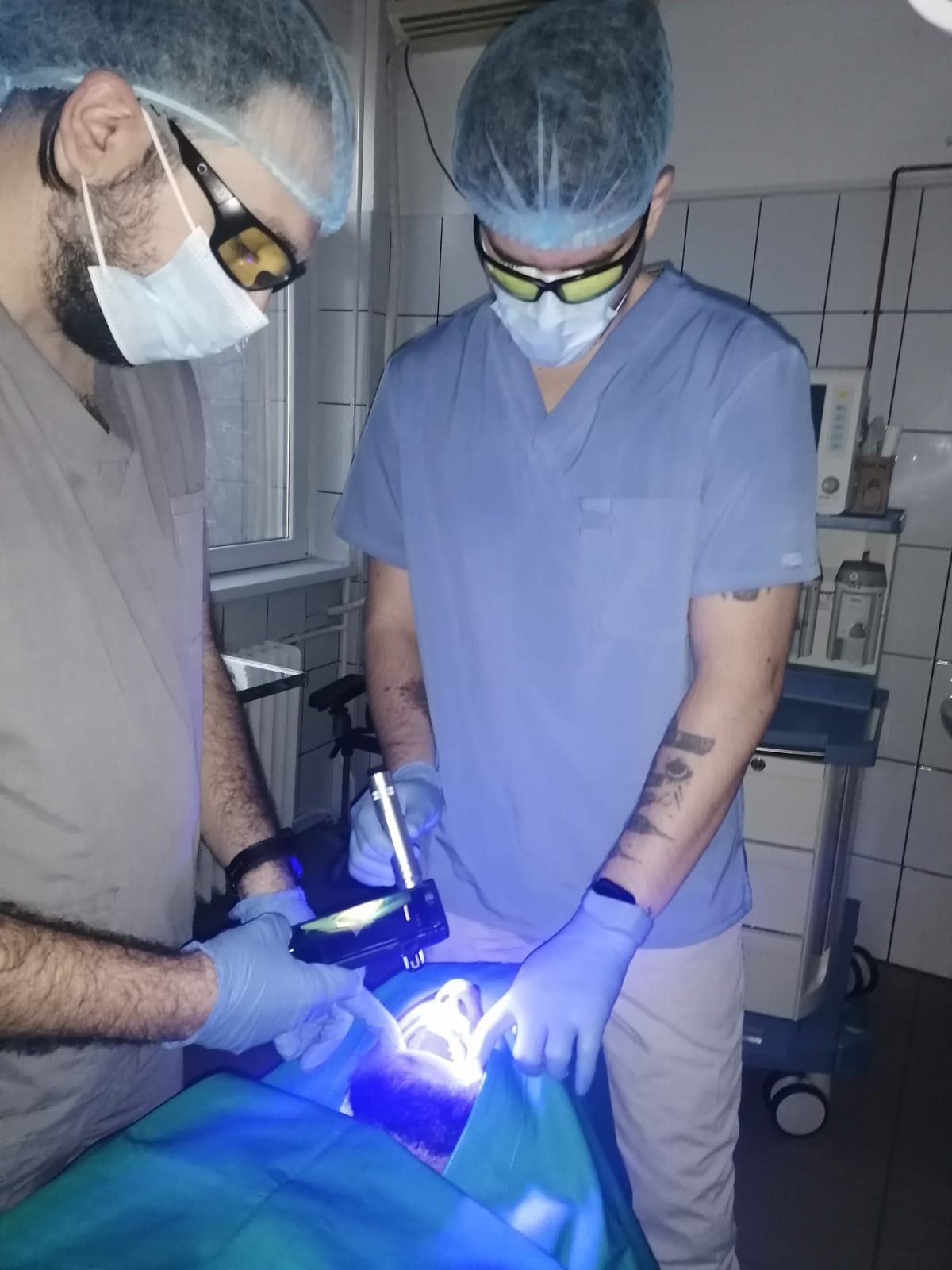

Supplement: Supplementary file 1 [file dentistry-13-00010-s001.zip › dentistry-3245039-supplementary/Medical Procedure Using OralID Technology2.jpeg]
